# Laser Melting of Prefabrication AlOOH-Activated Film on the Surface of Nodular Cast Iron and Its Associated Properties

**DOI:** 10.3390/ma16155486

**Published:** 2023-08-05

**Authors:** Xiaoyu Zhang, Xiuyuan Yin, Chen Liu, Changsheng Liu

**Affiliations:** 1 School of Materials Science and Engineering, Northeastern University, Shenyang 110819, China; 2 Key Laboratory for Anisotropy and Texture of Materials, Ministry of Education, Northeastern University, Shenyang 110819, China

**Keywords:** activated film, laser melting, sol–gel method, wear resistance, thermal stability

## Abstract

This study aimed to improve the absorption rate of laser energy on the surface of nodular cast iron and further improve its thermal stability and wear resistance. After a 0.3 mm thick AlOOH activation film was pre-sprayed onto the polished surface of the nodular cast iron, a GWLASER 6 kw fiber laser cladding system was used to prepare a mixed dense oxide layer mainly composed of Al_2_O_3_, Fe_3_O_4_, and SiO_2_ using the optimal laser melting parameters of 470 W (laser power) and 5.5 mm/s (scanning speed). By comparing and characterizing the prefabricated laser-melted surface, the laser-remelted surface with the same parameters, and the substrate surface, it was found that there was little difference in the structure, composition, and performance between the laser-remelted surface and the substrate surface except for the morphology. The morphology, structure, and performance of the laser-melted surface underwent significant changes, with a stable surface line roughness of 0.9 μm and a 300–400 μm deep heat-affected zone. It could undergo two 1100 °C thermal shock cycles; its average microhardness increased by more than one compared to the remelted and substrate surfaces of 300 HV, with a maximum hardness of 900 HV; and the average friction coefficient and wear quantity decreased to 0.4370 and 0.001 g, respectively. The prefabricated activated film layer greatly improved the thermal stability and wear resistance of the nodular cast iron surface while reducing the laser melting power.

## 1. Introduction

Nodular cast iron has better mechanical properties (plasticity and toughness) than gray cast iron, and its hardness is generally between 200 and 300 HV. Due to its low cost, good castability, mechanical properties, and other advantages, nodular cast iron has been widely used as a structural material for large mechanical parts, such as camshafts, crankshafts, and valve bodies [1,2]. However, the surface hardness and wear resistance of nodular cast iron is still low, and surface damage easily causes the failure of parts under high temperatures and wear [3], thus demanding frequent replacement of parts.

To improve the surface properties of nodular cast iron, researchers have used laser surface treatment technology to strengthen its surface and have achieved specific results. Kotarska et al. systematically investigated the laser alloying process of a titanium alloy on the surface of nodular cast iron and successfully improved its surface properties (2000 W laser power; 1.25 mm/s scanning speed). The surface hardness increased threefold, and the corrosion resistance improved significantly [4]. Janicki D et al. also prepared a titanium carbide (TiC)-reinforced layer on nodular cast iron via laser alloying (1500 W laser power; 1.25 mm/s scanning speed), which increased the surface hardness to 600 HV and effectively improved the wear resistance of the nodular cast iron surface. It has been proven that an increase in the TiC volume fraction will decrease the wear rate of a surface [5]. However, it is challenging to implement this due to technical problems and the high cost of laser alloying. Although this has effectively improved the surface properties of nodular cast iron, it is not easy to produce and apply on a large scale. On the other hand, Liu Ximing et al. made a nodular cast iron surface melt into white cast iron under the action of a laser via laser remelting technology (3500 W laser power; 0.2 mm/s scanning speed), which increased the surface hardness from 260 HV to 670 HV [6]. However, the white microstructure content produced by laser remelting seriously affects the high-temperature friction and corrosion resistance of nodular cast iron [7]. In recent years, Qu Weicheng et al. significantly increased the average microstructure hardness in the melting zone by repeated laser remelting, with a maximum of 870 HV (1400 W laser power; 5 mm/s scanning speed) and four times that of the base metal of the nodular cast iron sample [8]. However, the lack of surface characterization in their work made it impossible to determine the improvements in thermal stability and wear resistance. Fernández-Vicente et al. studied the effect of laser remelting on the surface microstructure of nodular cast iron from a practical point of view and obtained a 2.5 mm hardened layer via laser remelting and laser hardening; surface cracks were successfully reduced by adjusting the energy density [9]. Generally speaking, traditional laser remelting requires a smaller laser focus, higher laser power, or lower scanning speed to significantly increase the surface hardness of nodular cast iron. Additionally, it is also likely to affect surface thermal stability and wear resistance while improving hardness.

In practical applications, workpieces are usually machined on lathe machines and form bright surfaces, causing a low laser absorption rate and losses of energy during direct laser treatment. To improve absorption, pretreatment is essential. However, two challenges should be considered: the applicability of such pretreatment to complex workpiece surfaces and production cost. In addition, the surfaces of laser-treated nodular cast iron needs appropriate roughness. Whether the roughness is too large or small, further processing will be required, thereby increasing the cost and affecting the strengthened surface after the laser treatment. This leads to the fact that if the roughness of a nodular cast iron surface can be adjusted during the laser treatment of the surface, the subsequent processing steps can be omitted. Thus, losses in time, materials, and energy can be reduced. It is also possible to maximize the utilization of the strengthened surfaces with good usability generated by laser surface treatment.

As a result, Li Lei et al. proposed a new method for forming a ferroferric oxide (Fe_3_O_4_) film using laser flash melting after applying various kinds of prefabricated activated films on the surface of a steel material [10]; by taking advantage of the high hardness of Fe_3_O_4_ and the edge of oxidizing heredity, the surface properties of the iron and steel material were greatly improved, and the service life was effectively prolonged. The application of prefabricated activated film technology effectively enhances the laser absorption rate of cast iron surfaces, reduces the required laser power of surface laser melting, and shortens the processing time. However, relevant experiments have proven that the performance improvement in the surface characteristics of prefabricated laser melting is relatively low and that there are no issues related to surface roughness. Based on this, our project group tries to conduct further experiments on prefabricating activated films and comprehensively considers two laser surface treatment technologies, laser alloying and laser remelting, to improve this technology. While generating Fe_3_O_4_ at a flash rate on the surface, alumina (Al_2_O_3_) is introduced to form a dense protective film. A new approach that improves the thermal stability and wear resistance of nodular cast iron surfaces via laser melting after prefabricating activated films is proposed.

Based on this, boehmite (AlOOH) sol becomes the preferred material for prefabricating the newly activated films. As a high-quality material for preparing Al_2_O_3_, AlOOH has been widely used to prepare Al_2_O_3_ films with various complex shapes and excellent functions. Mauricio Gomez et al. used AlOOH to prepare mesoporous γ-Al_2_O_3_ films with a high specific surface area, which increased the specific surface area from 115 m^2^g^−1^ to 230 m^2^g^−1^ [11], significantly improving adsorption while ensuring the wear and corrosion resistance of the materials. Moreover, AlOOH can generate Al_2_O_3_ at high temperatures, which enables the activated film to rapidly develop Al_2_O_3_ via laser melting and successfully form a protective film on surfaces. In addition, AlOOH can be prepared by using an organic alkoxide solution and nitric acid as a superior peptizing agent [12], which enhances sol and nodular cast iron wettability and can perfectly replace the original ferric nitrate to form an absorption-enhancing activated film, thereby improving the surface laser absorption rate. Therefore, the present work tries to replace the original nitrate ferric salt with AlOOH sol with nitric acid as a peptizing agent. After prefabricating the activated film, laser melting is carried out to form an oxide film containing Al_2_O_3_ on the surface of nodular cast iron, improving the surface hardness, thermal stability, and wear resistance of the laser melting surface.

## 2. Materials and Methods

### 2.1. Prefabricated Activated Film

**Figure 1:**
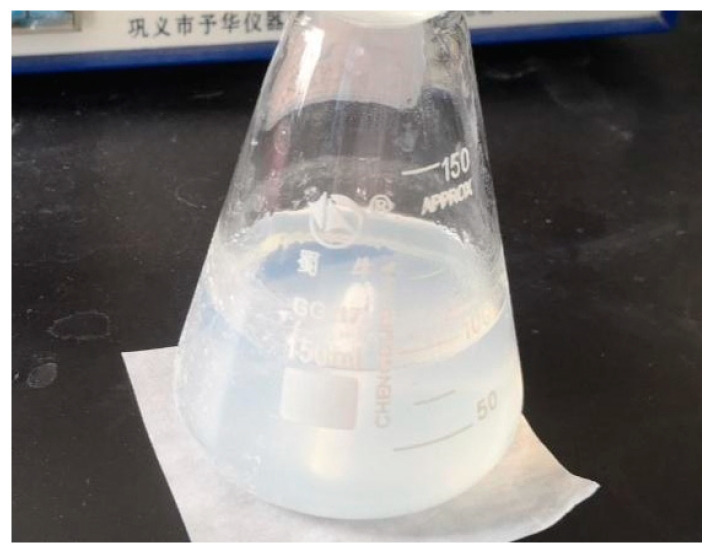
AlOOH sol after cooling.

AlOOH sol with high purity, large specific surface area, and a uniform particle size distribution was obtained via hydrolysis of aluminum isopropoxide using the sol–gel method [12,13]. As a solvent, ultrapure water and anhydrous ethanol were mixed at a volume ratio of 15:1, heated in a water bath at 85 °C, and then aluminum isopropoxide to water at a molar ratio of 1:75 was slowly added into the solvent in the water bath for hydrolysis. After 1.5 h of condensation reflux, a certain proportion of nitric acid was added to adjust the pH, and then condensation reflux was continued for 1 h to obtain a uniform and stable opalescent AlOOH sol, as shown in Figure 1.

**Figure 2:**
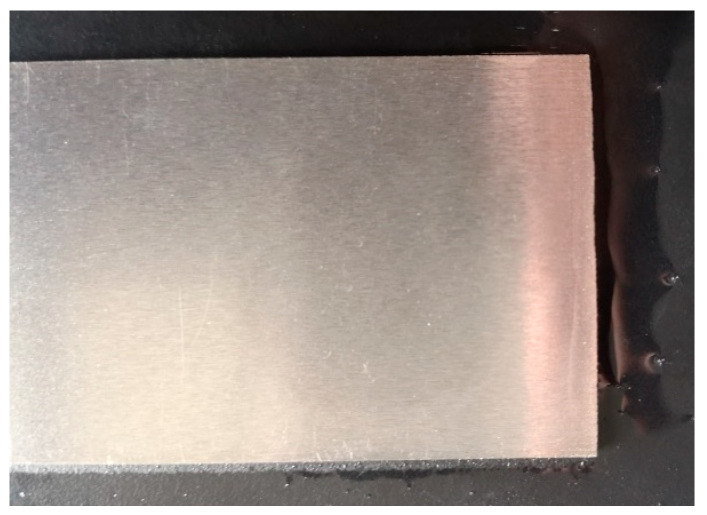
Surface morphology after spraying.

**Figure 3:**
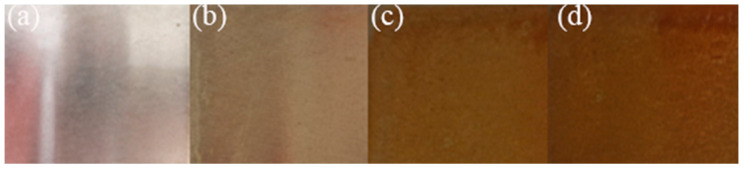
Surface color was recorded every 10 min after spraying. (**a**) 0 min; (**b**) 10 min; (**c**) 20 min; (**d**) 30 min.

After the AlOOH sol was cooled, it was evenly sprayed with a thickness of 0.3 mm onto the surface of the polished nodular cast iron blocks with ferrite matrix, as shown in Figure 2. The blocks were then left undisturbed to allow the prefabricated activation film to activate the surface. According to research by Li Lei et al., it was found that an acidic activation solution containing nitrate ions would form active ferrous nitrate and ferric nitrate activation films with Fe^2+^ and Fe^3+^ ions on the steel surface [10]; the surface of the nodular cast iron changed color over time from silver white to yellow–brown, as shown in Figure 3.

### 2.2. Laser Surface Treatment

**Figure 4:**
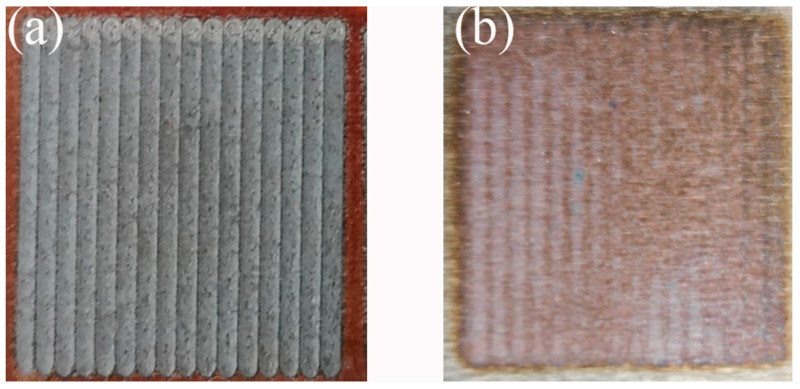
Macromorphology after laser surface treatment. (**a**) The prefabricated laser-melted surface; (**b**) the laser-remelted surface.

After successfully prefabricating and activating the polished surface of the nodular cast iron (about 20 min after spraying), a GWLASER 6 kw fiber laser cladding system was used for the laser melting experiments. The optimal parameters were obtained by measuring the overlap amount. The optimized parameters were as follows: 470 W laser power, 5.5 mm/s scanning speed, and 30% overlapping ratio. The control group was obtained by laser remelting the polished surface under the same conditions. Due to the low laser power and high laser reflectivity of the polished surface, the control group could not form a molten pool, as shown in Figure 4.

### 2.3. Microstructure and Performance Characterization

Through X-ray Diffraction (XRD) (BRUKER, D2 PHASER, Germany) using a copper target and the same scanning range (20–100°) as well as scanning speeds (step length 0.01°; step time 1 s), the phases for the polished surface of the nodular cast iron, the laser-remelted surface with the same parameters for the laser melting, and the prefabricated laser-melted surfaces were characterized. The morphology and composition of the three samples were characterized using an inverted metallographic microscope (OLYMPUS, GX71, Japan), laser confocal microscope (OLYMPUS, OLS3100, Japan), and scanning electron microscope (SEM) (Thermo Scientific, Apreo 2C, USA; backscattered electron probe: Oxford Instruments, SYMMETRY S2, UK; energy spectrum probe: Oxford Instruments, ULTIM MAX40, UK). A Thermal shock test (furnace temperature of 1100 °C, heat preservation for 10 min; flowing water at 20 °C, cooling for 10 min) was carried out in a tubular furnace to characterize the bonding and thermal stability of the three samples. The hardness and wear resistance of the three samples were characterized using a Vickers microhardness tester (Zhejiang South Ocean, MH-500, China) with the following parameters: a load of 1.96 N, maintained for 15 s, ten times for each sample, and a high-temperature fretting wear tester (Rtec-Instruments, MFT-5000, USA) with the following parameters: room temperature reciprocating friction, the upper sample is a Ø 6 mm silicon nitride ball, load of 3 N, speed of 2 cm/s, one-way direction of 4 mm, and a duration of 30 min. The wear trace morphology and composition of the three samples were characterized using a laser confocal microscope, SEM, and energy-dispersive spectroscopy (EDS) (Oxford Instruments, ULTIM MAX40, UK).

## 3. Results and Discussion

### 3.1. Phase Analysis of Surface

**Figure 5:**
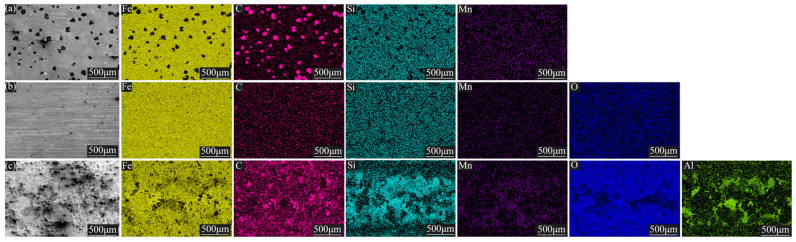
Surface scanning energy spectra. (**a**) The substrate surface; (**b**) the laser-remelted surface; (**c**) the prefabricated laser-melted surface.

**Figure 6:**
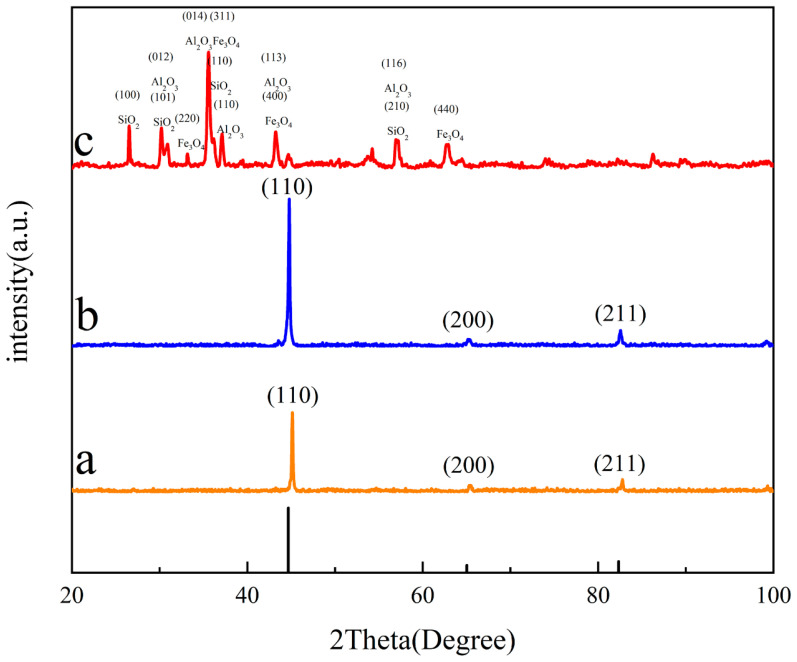
XRD atlas. (**a**) The substrate surface; (**b**) the laser-remelted surface; (**c**) the prefabricated laser-melted surface.

Three kinds of samples were prepared into block samples (10 × 10 × 5 mm) of an exact specification via wire cutting. By setting the SEM to a magnification of ×200, the EDS was used to scan the surface of the selected area and eliminated interference from trace elements, as shown in Figure 5. It was found that the substrate surface of the nodular cast iron mainly contained four elements, namely, Fe, C, Si, and Mn. Among them, C was primarily concentrated near the graphite balls, while the other elements were evenly distributed away from the graphite balls. Compared with the substrate surface, the laser-remelted surface had a lower C content and a higher O content, and the five main elements were evenly distributed on the surface. The O, C, Si, Mn, and Al contents on the prefabricated laser-melted surface increased significantly compared with those on the laser remelting surface. At the same time, the content of Fe decreased, in which O, C, Si, Mn, and Al were enriched considerably near the crescent top. XRD was used to characterize the phases on the surfaces of the three samples. The XRD patterns were obtained, as shown in Figure 6. The substrate surface showed an Fe phase with a slightly shifted diffraction peak. Compared with the substrate surface, the laser-remelted surface showed a small number of impurity peaks, and the diffraction peak shift disappeared, indicating a relatively standard Fe phase. The prefabricated laser-melted surface differed significantly from the first two, presenting a complex phase. According to the element distribution obtained by the EDS, it can be inferred that the surface shows a mixed phase with Al_2_O_3_, Fe_3_O_4_, and silicon dioxide (SiO_2_) as the main body.

According to the characterization results, the laser-remelted surface did not change significantly in composition. Although no apparent molten pool was produced during laser remelting, it was inferred that the graphite balls on the surface still escaped in the form of carbon dioxide under the action of the laser and played a role in repairing the surface. The holes and cracks derived from the disappearance of the graphite balls were repaired [14]. Due to the laser, all the elements were evenly distributed, and the surface was slightly oxidized, which elevated the O element content. The XRD spectrum showed trace impurity peaks, lattice distortion disappeared, diffraction peaks no longer shifted, and the surface presented an Fe phase with trace oxidation. The prefabricated laser-melted surface had a significant phase change due to the prefabricated AlOOH-activated film. After the laser melting, Al_2_O_3_, Fe_3_O_4_, and SiO_2_ were successfully formed on the surface. Additionally, the apparent molten pool effect affected the distribution of the surface elements. The prefabricated AlOOH-activated film was sintered at 500 °C to form Al_2_O_3_ [15]; thus, under the action of the high-energy laser beam, the prefabricated colloidal particles were sintered at a high speed in the molten pool to generate Al_2_O_3_, while the Fe in the molten pool was oxidized to create Fe_3_O_4_ under the induction of nitrate, nitrate provided O, and N escaped [10]. The Si in the substrate also combined with O in the molten pool to form SiO_2_ [16]. These three oxides were mixed to form a complex phase on the laser melting surface. Because of the movement of the molten pool, the light elements were on the top, and the heavy elements were on the bottom. Because the molten pool at the crescent top was more profound, the upward-floating elements of O, C, Si, Mn, and Al were increased. Under the movement of the molten pool, the light elements were enriched near the crescent top. Additionally, the Fe element was the opposite. As a result, microsegregation, which is common in laser melting, is produced [17].

### 3.2. Micromorphology

**Figure 7:**
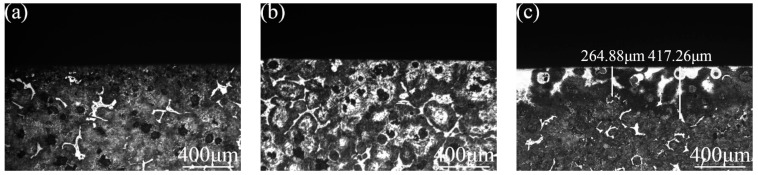
Section metallographic morphology. (**a**) Substrate surface; (**b**) laser-remelted surface; (**c**) prefabricated laser-melted surface.

**Figure 8:**
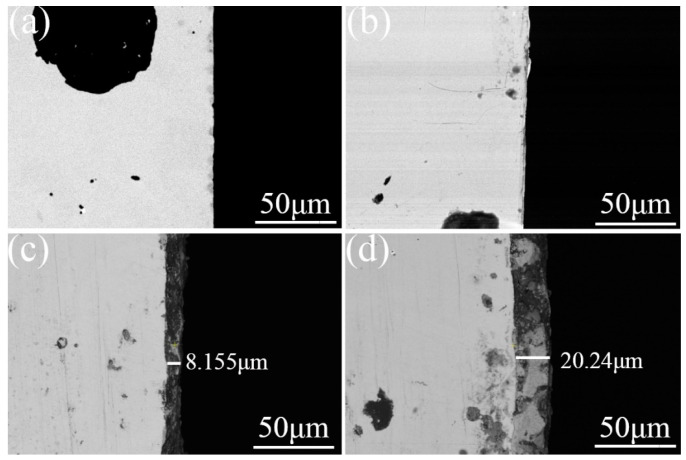
Backscattered electron imaging of section. (**a**) The substrate surface; (**b**) the laser-remelted surface; (**c**) the lap joint of the prefabricated laser-melted surface; (**d**) the crescent top of the prefabricated laser-melted surface.

The section structures of the three groups of samples were characterized using a metallographic microscope, as shown in Figure 7. Through characterization, it was found that the microstructures of the laser remelting and nodular cast iron sections were not significantly different. The section of the prefabricated laser-melted surface had a pronounced heat treatment area, about 300 μm (lap joint)–400 μm (crescent top) deep. Another group of three samples was taken for cross-section polishing; after polishing, they were characterized using an SEM backscattered electron probe, as shown in Figure 8. It was found that the surface morphology of the laser remelting samples and that of the nodular cast iron did not change. However, one mixed oxide layer of 10 μm (lap joint)–20 μm (crescent top) formed on the surface after the prefabricated laser melting.

If the activation film is not prefabricated, the laser action under the same power condition does not significantly change the surface structure of nodular cast iron. The polished surface reflects many lasers, the laser absorption rate is meager, and no apparent molten pool is generated. The repair of the laser beam and an increase in the O content only occur in the extremely thin area of the surface and do not penetrate deeply. However, laser melting after prefabricating the activated film obtains a 10–20 μm thick, complex oxide layer and a 300–400 μm deep, fine-grained zone. The oxide film composed of many light-element atoms confirms the phase characterization analysis, and a hard and brittle mixed oxide layer mainly composed of Al_2_O_3_, Fe_3_O_4_, and SiO_2_ indeed forms on the laser melting surface. Under the influence of high-energy laser beams, the thickness of the film layer shows a standard periodic change from the lap joint to the crescent top [18]. The heat-affected fine-grained zone shows an acicular martensite structure consistent with the structure state after the laser heat treatment [19]. This proves that the substrate absorbs the energy of the laser beam after the prefabricated activated film and the process of prefabricating the activation film dramatically improves the laser absorption rate of the polished surface of the nodular cast iron.

**Figure 9:**
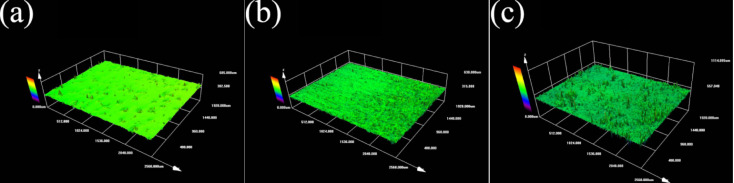
Surface 3D morphology. (**a**) Substrate surface; (**b**) laser-remelted surface; (**c**) prefabricated laser-melted surface.

**Figure 10:**
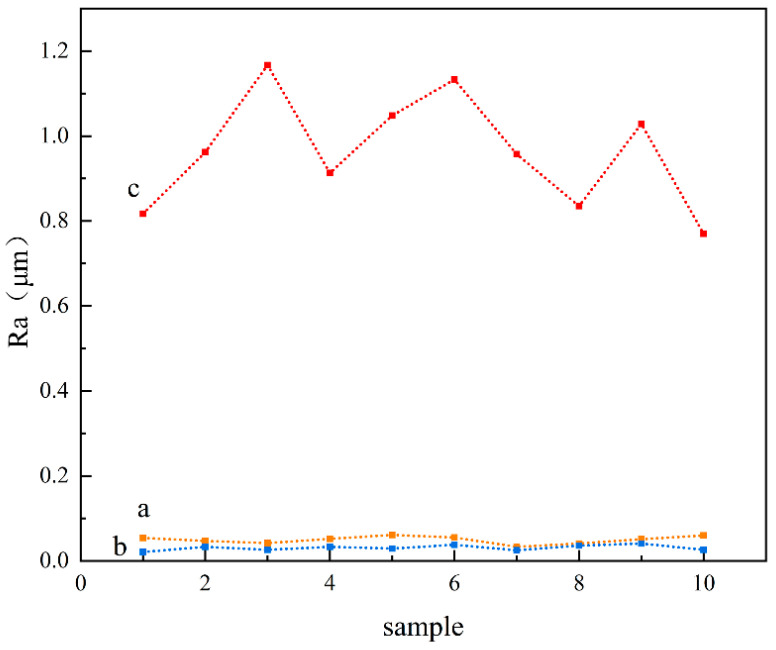
Surface line roughness (Ra) in the vertical laser treatment direction. (**a**) Substrate surface; (**b**) laser-remelted surface; (**c**) prefabricated laser-melted surface.

**Figure 11:**
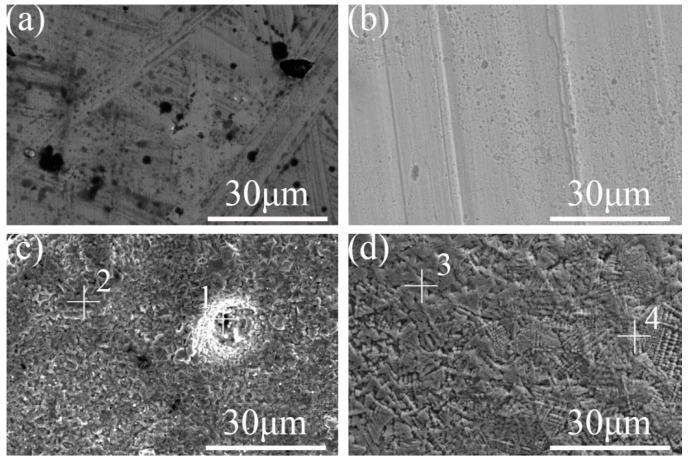
Surface micromorphology under SEM. (**a**) The substrate surface; (**b**) the laser-remelted surface; (**c**) the lap joint of the prefabricated laser-melted surface; (**d**) the crescent top of the prefabricated laser-melted surface.

**Figure 12:**
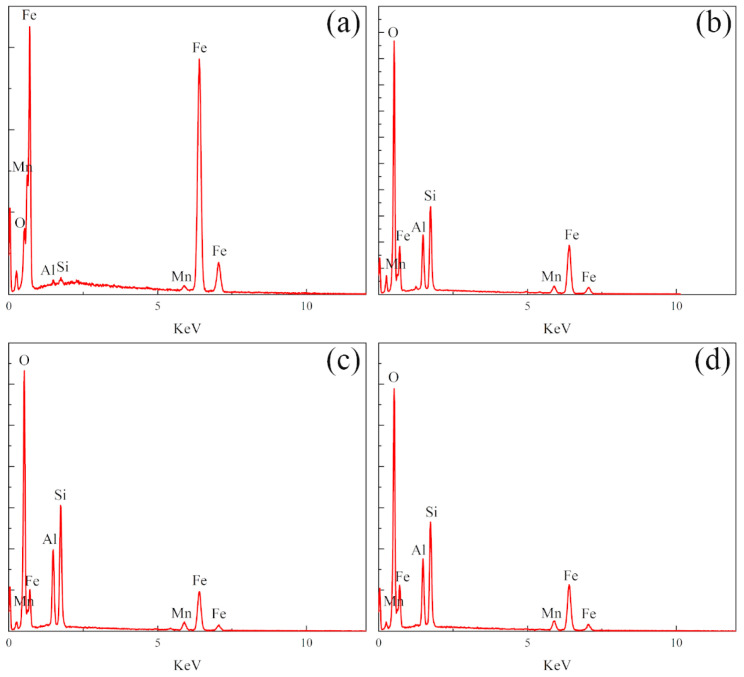
Energy spectra at points 1, 2, 3, and 4 in Figure 11. (**a**) Energy spectra of point 1; (**b**) energy spectra of point 2; (**c**) energy spectra of point 3; (**d**) energy spectra of point 4.

A laser confocal microscope was used to characterize the surface morphology of the three samples, and the 3D morphology of the three sample surfaces was obtained. The vertical *X*-axis direction was the direction of the laser treatment, as shown in Figure 9. It can be seen that the polished surface of the nodular cast iron is very flat as a whole, but there are deep pits. The laser-remelted surface, as a whole, shows transverse lines perpendicular to the laser processing direction, accompanied by small fluctuations on the surface. The prefabricated laser-melted surface showed the melting track morphology to be parallel to the laser processing direction. It has noticeable surface fluctuations, and the fluctuations at the lap joint are significantly more apparent than at the crescent top. Based on this, the surface line roughness (Ra) of the three groups of samples was measured with a sampling length of 2 mm in the direction perpendicular to the laser treatment, as shown in the diagram in Figure 10. It was seen that the surface line roughness (Ra) of the polished surface of the nodular cast iron was approximately 0.05 μm; the Ra of the laser remelting is stable at approximately 0.035 μm; however, the prefabricated laser melting improved and stabilized the surface line roughness in the range of 0.8–1.2 μm, with an average roughness Ra_(c)_ ≈ 0.9 μm. The SEM characterized the surface morphology of the three samples at a uniform 5000 times magnification, and the lap joint and crescent top of the prefabricated laser-melted surface were magnified and observed, respectively, as shown in Figure 11. It was found that, compared with the substrate surface with the distinct graphite balls and cast iron, the graphite balls on the laser-remelted surface were lost and showed a lamellar morphology perpendicular to the direction of the laser treatment. The complex surface morphology displayed by the surface melting track of the prefabricated laser-melted surface formed a dense and uniform dendritic crystal structure on the overall surface, with no macroscopic defects. Moreover, the dendritic crystal structure of the prefabricated laser-melted surface lap joint was smaller and denser, with more defects, large holes, and some spherical organization. An energy spectrum analysis was carried out on the places where the microstructure of the prefabricated laser-melted surface had apparent differences, as shown in Figure 12. The characterization results show that with the exception of the spheroid, which is mainly composed of Fe, the other dendritic structures, with slight differences, primarily comprise three oxygenates, namely, Al_2_O_3_, Fe_3_O_4_, and SiO_2_.

After polishing the surface of the nodular cast iron, the exposed graphite particles easily fell off, forming the surface morphology, as shown in Figure 9a. The surface line roughness (Ra) of the polished surface was slightly larger than that of the other alloys. Although laser remelting at this power only plays a role in repairing the surface, it still significantly changes the surface micromorphology. The surface line roughness (Ra) of the laser-remelted surface was reduced considerably under the laser repair, and the repair layer was oxidized to produce a reddish brown iron oxide under the laser, resulting in a lamellar structure perpendicular to the direction of the laser treatment. The prefabricated laser-melted surface formed an evident molten pool. The laser-melted surface line roughness increases significantly under the internal movement of a molten pool and laser beam stirring [20]. The surface roughness after laser surface treatment has a special relationship with the amount of overlap [21]. The surface roughness is mainly controlled by the laser power and speed when the surface composition and the size of the laser spot remain unchanged. This experiment successfully maintained the line roughness (Ra) of the prefabricated laser-melted surface at 0.8–1.2 μm. The surface of the molten pool showed a new micromorphology after solidification. The prefabricated laser-melted surface comprised a dense dendrite structure formed by mixing Al_2_O_3_, Fe_3_O_4_, and SiO_2_ oxides as the main body, creating a dense surface. The dendritic structure was prepared through the ultra-high-temperature gradient and solidification rate induced by the laser [22]. It was smaller than the dendrite in orbit and had more defects [23].

### 3.3. Thermal Stability and Wear Resistance

**Figure 13:**
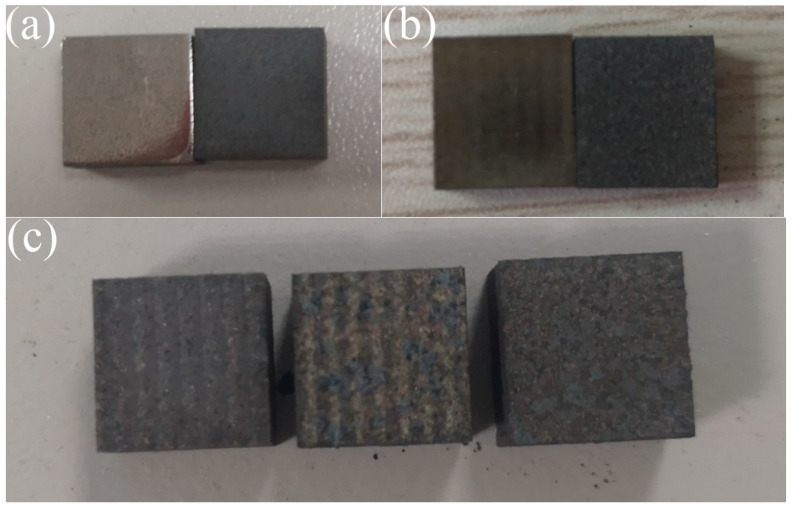
Surface morphology of samples after thermal shock. (**a**) Substrate surface; (**b**) laser-remelted surface; (**c**) prefabricated laser-melted surface.

A thermal shock test of the sample was carried out in a tubular furnace to characterize the bonding and thermal stability of the laser melting surface. The characterization results show that the polished surface and laser-remelted surface of the nodular cast iron were oxidized entirely after one thermal shock cycle, and the surface thermal stability was poor. The pre-oxidized layer could be wholly peeled off after two thermal shock cycles for the 10–20 μm thick pre-melted surface. The laser-melted surface had good adhesion and significantly improved thermal stability, as shown in Figure 13. The Fe phase of the polished surface of the nodular cast iron was directly exposed. Although the texture was uniform and the bonding was good, it was easy to oxidize at high temperatures. Because the laser-remelted surface was oxidized by laser microlayer repair, the surface was generally reddish brown, mostly composed of the ferric oxide phase. Wire cutting leads to edge oxidation and blackening, with poor thermal stability. The prefabricated laser melting oxide layer is a complex oxide layer formed by molten pool solidification. It binds well to the substrate because of the laser action [18]. From the above characterization of the pre-oxidized layer, it was seen that the surface morphology of the pre-oxidized layer was dense, with few defects. Furthermore, the oxide layer, mainly composed of Al_2_O_3_, Fe_3_O_4_, and SiO_2_ oxides, had good antioxidant capacity [24,25,26]. Therefore, it has good thermal stability.

**Figure 14:**
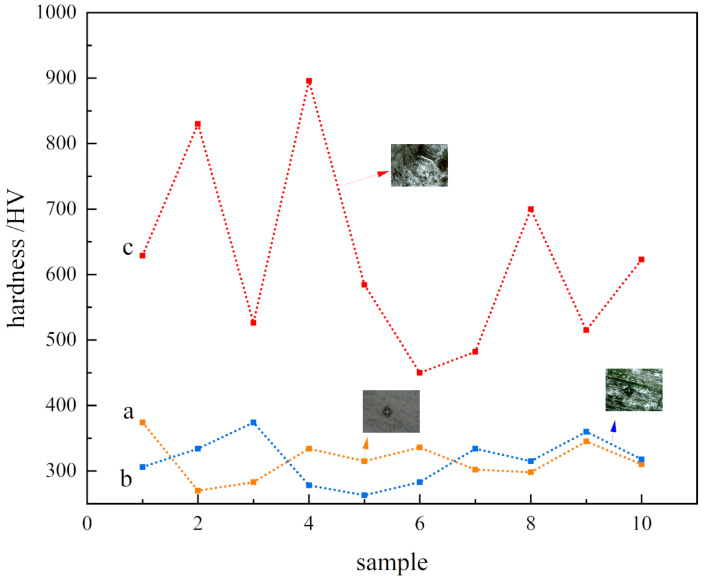
Microhardness and indentation size. (**a**) Substrate surface; (**b**) laser-remelted surface; (**c**) prefabricated laser-melted surface.

A micro-Vickers hardness tester was used to characterize the surface hardness and conduct an indentation test on the three sample surfaces (indentation depth can be estimated by calculating indentation size to ensure that the measured indentation does not penetrate the surface oxide layer). The results are shown in Figure 14. It was seen that the microhardness of the polished surface of the nodular cast iron and the laser-remelted surface had not changed, with both at about 300 HV. However, the hardness of the prefabricated laser-melted surface significantly improved, the indentation dramatically reduced, and the hardness value was discrete due to the large surface roughness. It was necessary to take several measurements to summarize the results. According to the results, the average microhardness increased by about one, and the highest hardness reached 900 HV. It was seen from the abovementioned characterization that the surface microstructure and structure did not change much after the laser remelting, and the repair oxide layer was too thin to measure; thus, the microhardness of the laser-remelted surface measured by the hardness tester exhibited little change. The prefabricated laser-melted surface formed with a thickness of 10–20 μm on the surface for the dense oxide layer. The surface roughness is significant, resulting in a relatively discrete hardness value. At the same time, due to the high hardness of the Al_2_O_3_, Fe_3_O_4_, and SiO_2_ oxide mixtures on the laser melting surface, a dense dendritic-enhanced phase surface was present, resulting in a significant increase in surface microhardness [27].

**Figure 15:**
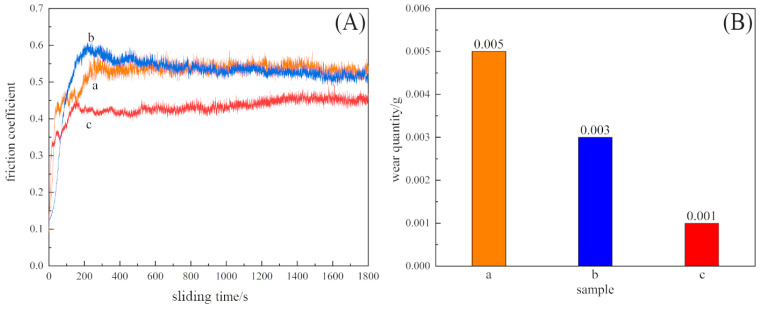
Variation in friction coefficient with time (**A**) and wear quantity (**B**). (**a**) The substrate surface; (**b**) laser-remelted surface; (**c**) prefabricated laser-melted surface.

**Figure 16:**
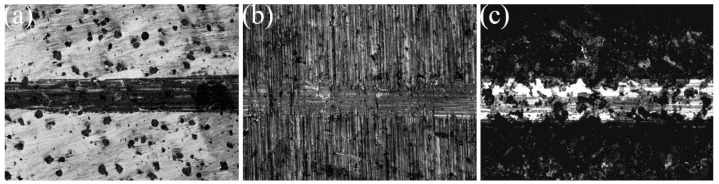
Wear traces under laser confocal observation. (**a**) Substrate surface; (**b**) laser-remelted surface; (**c**) prefabricated laser-melted surface.

**Figure 17:**
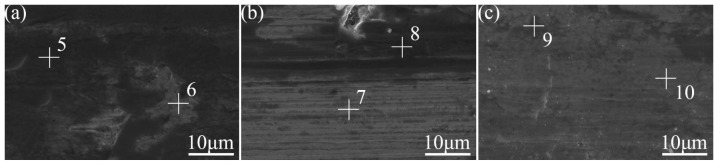
Wear micromorphology under SEM. (**a**) Substrate surface; (**b**) laser-remelted surface; (**c**) prefabricated laser-melted surface.

**Figure 18:**
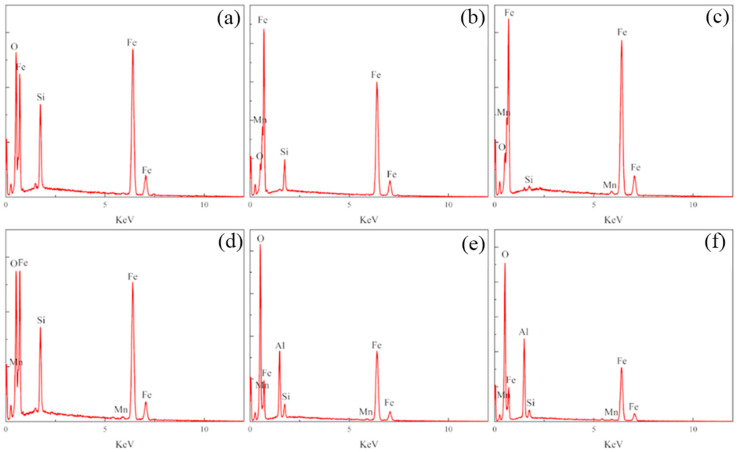
Energy spectra at points 5, 6, 7, 8, 9, and 10 in Figure 17. (**a**) Energy spectra of point 5; (**b**) energy spectra of point 6; (**c**) energy spectra of point 7; (**d**) energy spectra of point 8; (**e**) energy spectra of point 9; (**f**) energy spectra of point 10.

The surface wear resistance of the three samples was characterized, and the real-time friction coefficient as well as the mass wear amount after the experiment were measured, as shown in Figure 15. The friction coefficient of the laser-remelted surface and the polished surface of the nodular cast iron showed little difference, with both being around 0.5280, and the volume loss decreased from 0.005 g to 0.003 g. The prefabricated laser-melted surface reduced the friction coefficient to 0.4370 and the volume loss to 0.001 g, significantly improving the wear resistance. A laser confocal microscope was used to observe the overall morphology of the three kinds of wear traces, as shown in Figure 16. SEM was used to magnify the internal morphology of the wear traces at 8000 times magnification, as shown in Figure 17. It was seen that there is no furrow inside the wear traces of the nodular cast iron, but that there are pits and two different degrees of contrast simultaneously. Furthermore, there are conspicuous furrows and holes in the wear trace of the laser remelting as well as two different contrast degrees. Moreover, less apparent furrows and very obvious layered protrusions inside the laser melting grinding marks, without any pits, can be seen, with two areas of different contrast degrees present. An energy spectrum dot analysis was carried out for places with different contrasts, as shown in Figure 18. The results show that the difference in element content at points 5 and 6 is mainly reflected by the difference in the proportion of O. The difference in element contents between points 7 and 8 is also primarily reflected by the difference in the O and Si content ratio. The element content difference between points 9 and 10 is minimal.

From the characterization, it was seen that the wear behavior of the polished surface of the nodular cast iron is mainly adhesive wear. At the same time, the natural peeling of the graphite ball causes fatigue wear. When friction wear occurs on the surface of nodular cast iron, it should be run in first; then, a stable friction coefficient would be maintained for friction wear. At the same time, adhesive wear occurs inside the wear trace. This is oxidized due to the heat generated by friction and wear to protect the substrate [28]. At this time, uneven oxidation leads to different contrast degrees. Because the substrate material is soft, the graphite ball is easy to peel off, and the wear resistance is poor. The wear behavior of the laser-remelted surface changes, mainly due to abrasive wear and accompanied by a small amount of fatigue wear. The laser-remelted surface also runs in first when carrying out friction and wear. After running in, the friction coefficient of nodular cast iron slowly decreases from a high to an average value. The wear amount is also reduced because the laser-remelted surface is oxidized and repaired by the laser. A thin layer of oxide and white cast iron on the surface plays a protective role in the early stage [8,29], reducing the impact of graphite balls peeling and causing the wear behavior to change to mainly abrasive wear. Different contrast degrees were produced during wear due to the uneven oxidation and the distribution of the Si element inside the wear trace. A small amount of abrasive wear accompanied the adhesive wear behavior of the prefabricated laser melting surface. The lamellar structure about to be worn off can even be observed from the worn scar surface. The prefabricated laser-melted surface maintained a low friction coefficient after running in, indicating that the pre-oxidized surface was smoother than the cast iron following this process. At the same time, the wear quantity of the prefabricated laser-melted surface was significantly reduced, although the surface roughness was large. Measuring the wear volume is difficult, but the surface wear resistance was effectively improved. This is because the prefabricated laser-melted surface conforms to Archard’s law: the higher the surface hardness is, the better the wear resistance is [30]. The different content of the various oxides mainly caused the contrast degrees in the wear trace. It was seen from the aforementioned thermal stability analysis that the oxide layer of the prefabricated laser-melted surface was not oxidized during the friction and wear process and that different contrast degrees are produced during the preparation.

**Figure 19:**
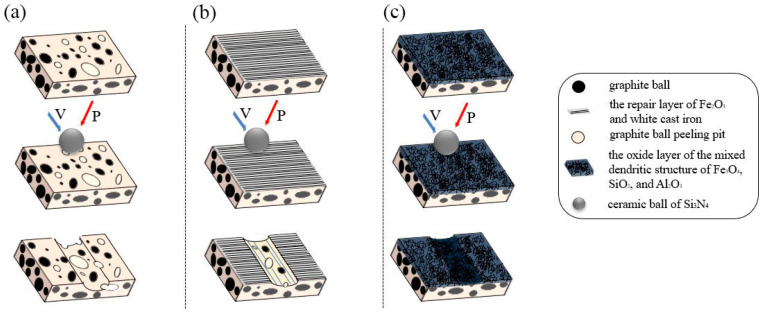
Schematic diagram of the wear mechanism of the surface. (**a**) Substrate surface; (**b**) laser-remelted surface; (**c**) prefabricated laser-melted surface.

According to the above characterization, the wear resistance of the prefabricated laser-melted surface improved to a certain extent. The above characterization analysis is summarized to refine the mechanism of improving the wear resistance of the prefabricated laser melting surface, as shown in Figure 19. According to the various characterizations of the nodular cast iron surface, laser remelting surface, and prefabricated laser melting surface, the Fe phase of the nodular cast iron is exposed, and the graphite balls on the surface are straightforward to peel off. The surface presents a cratered and soft Fe phase. Under the friction of the upper sample, adhesive wear occurs, and large pieces of tissue peel off as the upper sample moves while driving the internal graphite balls to peel off together. After the laser remelting of the nodular cast iron with the above experimental parameters, a skinny lamellar repair layer forms on the surface of the nodular cast iron [8], the surface pits are repaired [14], and the substrate is protected [29]. However, the repair layer is very thin, so it is susceptible to wearing out under abrasive wear, and it may revert to a situation wherein the nodular cast iron substrate is directly rubbed with the sample. This prefabricated laser-melted surface technology effectively improves the laser absorption rate by prefabricating the AlOOH-activated film and forms a prominent oxide layer with a stable surface roughness of 470 W. The oxide layer comprises mixed dendrites mainly composed of Al_2_O_3_, Fe_3_O_4_, and SiO_2_. The complex oxide layer with a dense structure has a high hardness [27], successfully protecting the graphite ball of the substrate. The fatigue wear caused by graphite ball peeling is effectively avoided, and the wear resistance of the surface is significantly improved [30].

## 4. Conclusions

The following conclusions can be made:

1. The prefabricated AlOOH-activated film changes the surface composition after laser melting, and a dense oxide layer containing Al_2_O_3_, Fe_3_O_4_, SiO_2_, and other oxides appears on the surface of the nodular cast iron after laser melting.
2. The prefabricated AlOOH-activated film improves the laser absorption rate of the treated surface. After the AlOOH sol containing nitric acid is coated on the polished surface of nodular cast iron, the yellow–brown prefabricated film generated by the reaction significantly improves the laser absorption rate. The best performance laser-melted surface can be obtained under laser parameters of 470 W and 5.5 mm/s.
3. The laser-melted surface after prefabrication with the AlOOH-activated film exhibits good bonding with the substrate and effectively improves the thermal stability of the nodular cast iron surface. It can effectively resist two thermal shock cycles at 1100 °C.
4. The prefabricated AlOOH-activated laser-melted surface film has a stable surface line roughness, with an average of 0.9 μm. It improves the surface microhardness, with an average increase by nearly one, reaching a maximum of 900 HV, effectively improving the wear resistance of the nodular cast iron surface and reducing the friction coefficient by almost 20% and the wear quantity by one-fifth.

## Data Availability

Not applicable.

## References

[1] Wang J.G., Wang Z.Q., Ren S.A. (2020). The effect of Ti addition on microstructure and mechanical properties of nodular cast iron. J. Jilin Univ. (Eng. Ed.).

[2] Nadal R.L., Roca A.S., Fals H.D.C., Zoqui E.J. (2015). Mechanical properties of thermoformed hypoeutectic gray cast iron. J. Mater. Process. Technol..

[3] Zhu L., Liu Y., Li Z., Zhou L., Li Y., Xiong A. (2020). Microstructure and properties of Cu-Ti-Ni composite coatings on gray cast iron fabricated by laser cladding-ScienceDirect. Opt. Laser Technol..

[4] Kotarska A. (2021). The Laser Alloying Process of Ductile Cast Iron Surface with Titanium. Metals.

[5] Janicki D. (2020). The friction and wear behavior of in-situ titanium carbide reinforced composite layers manufactured on ductile cast iron by laser surface alloying. Surf. Coat. Technol..

[6] Liu X.M., Lian J.S. (2002). Microstructure and properties of ferritic nodular cast iron after laser surface remelting. J. Mater. Heat Treat..

[7] Lu S.S., Zhou J.S., Wang L.G., Liang J. (2022). Corrosion resistance and high-temperature tribological properties of laser cladding Ni-Co composite coating on nodular cast iron. China Surf. Eng..

[8] Qu W.C. (2017). Thesis Research on Laser Remelting Process of Nodular Cast Iron Surface. Master’s Thesis.

[9] Fernández-Vicente A., Pellizzari M., Arias J.L. (2012). Feasibility of laser surface treatment of pearlitic and bainitic ductile irons for hot rolls. J. Mater. Process. Technol..

[10] Li L. (2023). Laser Flash Forming Method of Oxide Film on Steel Surface at High Temperature.

[11] Mauricio G., Pizarro J., Castillo X., Díaz C., Ghisolfi A., de Lourdes Chávez M., Cazorla-Amorós D., Arenas-Alatorre J. (2021). Preparation of mesoporous γ-Al_2_O_3_ with high surface area from an AlOOH extract of recycling biomass ash. J. Environ. Chem. Eng..

[12] Ren Y.R. (1989). Structure, properties, and applications of aluminum sol. Inorg. Salt Ind..

[13] Duan N., Zhang X.T., Lu C.L., Zhang Y.F., Li C.R. (2021). Effect of nitric acid on the particle size of AlOOH gel prepared by sol-gel method. Silic. Bull..

[14] Ma S., Zhou T., Zhou H., Chang G., Zhi B., Wang S. (2020). Bionic Repair of Thermal Fatigue Cracks in Ductile Iron by Laser Melting with Different Laser Parameters. Met.-Open Access Metall. J..

[15] Duan N., Zhang X.T., Lu C.L., Zhang Y., Li C., Xiong J. (2022). Effect of rheological properties of AlOOH sol on the preparation of Al_2_O_3_ nanofiltration membrane by sol-gel method. Ceram. Int..

[16] Liu S.F., Xiao W.C., Kai J.-J., Yang T., Xiao B., Ju J., Zhou Y., Zhao Y., Jiao Z., Luan J. (2023). Simultaneously Improving Mechanical Properties and Oxidation Resistance of Ti-bearing High-entropy Superalloys at Intermediate Temperature via Silicon Addition. J. Mater. Sci. Technol..

[17] Yang H., Jing G., Gao P., Wang Z., Li X. (2020). Effects of circular beam oscillation technique on formability and solidification behaviour of selective laser melted Inconel 718: From single tracks to cuboid samples. J. Mater. Sci. Technol..

[18] Wang L.X., Huang Y.M., Yuan Y.X., Jia C., Yang L. (2022). Microstructure, wear and oxidation resistance of Al-doped Ti–Si_3_N_4_ coatings by laser cladding. Surf. Coat. Technol..

[19] Boccardo A.D., Catalán N., Celentano D.J., Ramos-Moore E. (2021). A Thermo-metallurgical Model for Laser Surface Engineering Treatment of Nodular Cast Iron. Metall. Mater. Trans. B.

[20] Atefe R., Farzin G., Farshid M. (2023). Microstructural Characteristics and Tribological Properties of the Localized Laser Surface Treatment of AISI 420 Stainless Steel. Tribol. Int..

[21] Yan Q., Yang K., Wang Z.D., Chen M.Z., Sun G.F., Ni Z.H. (2022). Surface roughness optimization and high-temperature wear performance of H13 coating fabricated by extreme high-speed laser cladding. Opt. Laser Technol..

[22] Gu D., Shen Y., Lu Z. (2009). Microstructural characteristics and formation mechanism of direct laser-sintered Cu-based alloys reinforced with Ni particles. Mater. Des..

[23] Cao Y., Zhu P., Yang Y., Shi W., Qiu M., Wang H., Xie P. (2022). Dislocation Mechanism and Grain Refinement of Surface Modification of NV E690 Cladding Layer Induced by Laser Shock Peening. Materials.

[24] Shaghayegh S., Seyed M.M., Hamidreza R., Yaser V., Stefano T., Filippo B. (2023). Improving Thermal Stability and Textural Properties of Mesoporous γ-alumina Granules by Zr-La Dopants. J. Alloys Compd..

[25] Huang X., Gao Y., Li Q., Jian Y., Xiao P., Li B., Wang Y., Yi Y., Zhao S. (2021). Effect of Si element on improving the oxidation resistance of hybrid (Ti_5_Si_3_ + TiC) particles reinforced Ti_6_Al_4_V matrix composites. Corros. Sci..

[26] Dai H.C.C. (2017). Microstructure and properties of Ti-Al coating and Ti-Al-Si system coatings on Ti-6Al-4V fabricated by laser surface alloying. Surf. Coat. Technol..

[27] Tyagi R., Das A.K., Mandal A. (2021). Formation of superhydrophobic surface with enhanced hardness and wear resistance by electrical discharge coating process. Tribol. Int..

[28] Zhou J.Z., Wang J.J., Feng X., Meng X.K., Xu J. (2016). Tribological Behavior of Laser Textured Nodular Cast Iron Surface. Chin. J. Lasers.

[29] Camurri C., Maril J., Romero E. (2021). Effect of the Morphology, Size, Distribution and Homogeneity of Carbides and Matrix on Wear Resistance in High Cr-Alloys White Cast Iron. Mater. Sci. Forum.

[30] Reichelt M., Cappella B. (2021). Large scale multi-parameter analysis of wear of self-mated 100Cr_6_ steel—A study of the validity of Archard’s law. Tribol. Int..

